# New 2-Phenylthiazoles as Potential Sortase A Inhibitors: Synthesis, Biological Evaluation and Molecular Docking

**DOI:** 10.3390/molecules22111827

**Published:** 2017-10-27

**Authors:** Smaranda Dafina Oniga, Cătălin Araniciu, Mariana Doina Palage, Marcela Popa, Mariana Carmen Chifiriuc, Gabriel Marc, Adrian Pirnau, Cristina Ioana Stoica, Ioannis Lagoudis, Theodoros Dragoumis, Ovidiu Oniga

**Affiliations:** 1 Faculty of Pharmacy, “Iuliu Hațieganu” University of Medicine and Pharmacy, 8 Victor Babes St, 400012 Cluj-Napoca, Romania; smaranda.oniga@umfcluj.ro (S.D.O.); Marc.Gabriel@umfcluj.ro (G.M.); Stoica.Cristina@umfcluj.ro (C.I.S.); lagoudisjohn@yahoo.gr (I.L.); theodorosdragoumis@icloud.com (T.D.); ooniga@umfcluj.ro (O.O.); 2 Department of Microbiology, Faculty of Biology, University of Bucharest, 1-3 Portocalelor Street, 60101 Bucharest, Romania; bmarcelica@yahoo.com (M.P.); carmen_balotescu@yahoo.com (M.C.C.); 3 Research Institute of the University of Bucharest-ICUB, 91-95 Independentei Street, 050095 Bucharest, Romania; 4 National Institute for Research and Development of Isotopic and Molecular Technologies, 67-103 Donat Street, 400293 Cluj-Napoca, Romania; apirnau@yahoo.com

**Keywords:** 2-phenylthiazole, thiazole, antimicrobial, anti-biofilm activity, Sortase A inhibitor, enteroccocus

## Abstract

Sortase A inhibition is a well establish strategy for decreasing bacterial virulence by affecting numerous key processes that control biofilm formation, host cell entry, evasion and suppression of the immune response and acquisition of essential nutrients. A meta-analysis of structures known to act as Sortase A inhibitors provided the starting point for identifying a new potential scaffold. Based on this template a series of new potential Sortase A inhibitors, that contain the 2-phenylthiazole moiety, were synthesized. The physicochemical characterisation confirmed the identity of the proposed structures. Antibacterial activity evaluation showed that the new compounds have a reduced activity against bacterial cell viability. However, the compounds prevent biofilm formation at very low concentrations, especially in the case of *E. faecalis*. Molecular docking studies performed estimate that this is most likely due to the inhibition of Sortase A. The new compounds could be used as add-on therapies together with known antibacterial agents in order to combat multidrug-resistance enterococcal infections.

## 1. Introduction

Preventing antibiotic resistance and identifying new antibiotics that are active against the multidrug resistant bacteria are major current concerns [1,2]. Particularly, Gram-positive bacteria are common causes of nosocomial bloodstream and other infections. Methicillin-resistant *Staphylococcus aureus* (MRSA) and vancomycin-resistant enterococci (VRE) are the most imperative concern [3].

Gram-positive bacteria have a thick cell wall that surrounds the plasma membrane. This cell wall is composed of peptidoglycan that serves as a matrix for the covalent attachment of wall teichoic acid, surface proteins and polysaccharide capsule [4,5]. Sortases are cysteine transpeptidases that are able to join proteins bearing an appropriate sorting signal to strategically positioned amino groups on the cell surface. Sortases are not essential for bacterial viability, but they become important virulence factors because the surface proteins that they help display mediate biofilm formation, bacterial adhesion to host tissues, host cell entry, evasion and suppression of the immune response and acquisition of essential nutrients [6].

From the study of the nearly a thousand sortase homologues identified so far in different Gram-positive bacterial strains it is apparent that they share similar structures, catalytic attributes and substrates. The prototypical Sortase A enzyme, the best understood and most studied, is the one from *S. aureus* (Sa-SrtA). At least twenty different *S. aureus* surface proteins that carry a C-terminal LPXTG (leucine, proline, X, theronine, and glycine, where X is any amino acid) motif have been described as being anchored via Sa-SrtA. Some of the LPXTG surface proteins, including Spa, FnbpA, FnbpB, ClfA, ClfB and other microbial surface components recognizing adhesive matrix molecules (MSCRAMMs), are directly linked with biofilm formation [7,8,9,10,11]. Bacterial ability to form biofilms allows them to colonize inert substrates (e.g., implantable medical devices) thus causing infections that are difficult to treat because the biofilm matrix and phenotypic characteristics of the bacteria confer resistance to both the host immune response and the action of antimicrobial drugs [9,12,13,14,15].

Selecting Sortase A as a target for the development of new antibacterial drugs is supported primarily by the key role this enzyme has in Gram-positive bacterial virulence and also by the fact that no sortase homologs are present in eukaryota, which could lead to specific toxicity against bacteria with a good pharmaco-toxicological profile in humans. In the same time, the fact that these enzymes are found in the membrane/cell-wall implies that inhibitors do not have to penetrate inside the bacterial cell, which diminishes the pharmacokinetic requirements for a new inhibitor. Another potential benefit of Sortase A inhibitors would be the fact that they will act only by reducing virulence and biofilm formation, thus exerting no selective pressure that could eventually lead to development of resistant bacteria strains [14,16,17].

The effort to discover a Sortase A inhibitor has identified various structural scaffolds as potentially clinical useful solutions. These include: 2-phenylbenzofuran-3-carboxamide [18], 2-phenylbenzo[*d*]oxazole-7-carboxamide [19], 2-(2-phenylhydrazinylidene)alkanoic acids [20], benzisothiazolinone [21], indolethiazolidine [22], rhodanines [23], diarylacrylonitriles, triazolo-thiadiazole [24], and other structures as presented in the reviews of these fields [16,25,26].

Although many of the chemical classes rely on covalent interactions with the active site some have proved to have a non-covalent inhibition process [25,27]. Based on our previous research experience [28,29,30,31] and considering the molecular scaffolds used by other research groups and the general pharmacophore requirements described in the computational studies [23,25,32] we decided to synthesize a series of 2-phenyl-thiazole derivatives.

**Figure 1:**
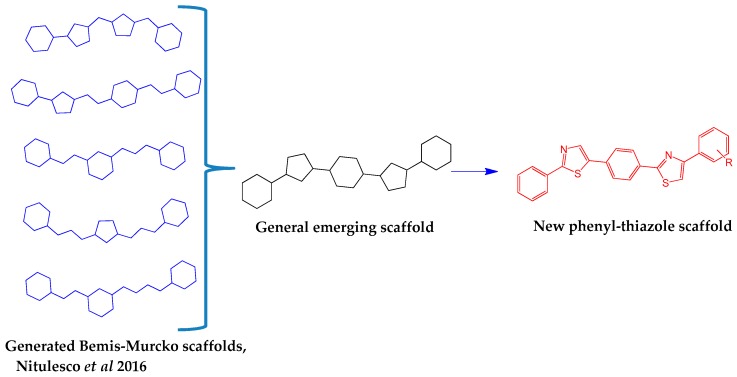
The design strategy for the target compounds.

The proposed structures are in agreement with the Bemis-Murcko scaffolding results obtained by the analysis of 156 small compounds that are known inhibitors of Sortase A [25]. The new compounds have a scaffold characterised by five aromatic rings linked directly which allows for limited free rotation of the bonds, as shown in Figure 1. This ensures low molecular flexibility with a number of rotatable bonds less or equal to 4 thus complying with the most relevant descriptor for SrtA affinity [25]. The requirement for the presence of at least two hydrophobic regions, with molecular LogP values above 2, is also met by this scaffold. The inclusion of the two thiazole rings within the scaffold ensures the potential for hydrogen bond formation with 2 or 3 hydrogen bond acceptor atoms [23,25].

In order to validate the biological potential of the synthesized structures as Sortase A inhibitors we performed a biological determination of the antibacterial and anti-biofilm effect. If the new compounds act by inhibiting sortases, cell viability should not be influenced, while the ability to colonize inert substrates by biofilm formation should be heavily decreased. The hypothesis of sortase inhibition was further verified by a series of molecular docking studies to estimate the binding potential and mode of the new synthesized compounds to different subtypes of Sortase A (e.g., sortase from *S. aureus* and *E. faecalis*).

## 2. Results and Discussion

### 2.1. Chemistry

**Figure 2:**
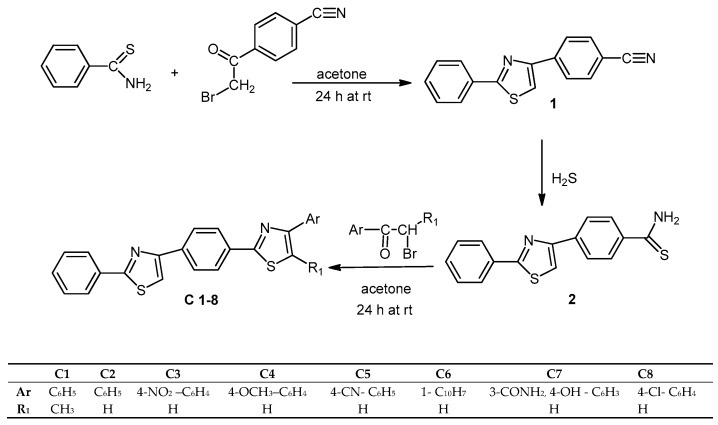
The synthesis of the 2-phenylthiazole derivatives (**C1**–**8**).

Using the reaction route described in Figure 2, eight final compounds were obtained. The two key intermediates, the nitrile derivative **1** and the thioamide derivative **2**, were also characterized in order to establish identity and purity. The thioamide moiety in intermediate **2** allowed for various subsequent Hantzsch condensations with α-bromocarbonyl compounds that yielded a new thiazole ring.

All structural analysis data proved that the structures of the obtained compounds are in accordance with the proposed structures. The ^1^H-NMR spectra of compounds **C1**–**8** showed the disappearance of the 2H singlet from the thioamide intermediate **2** and the presence of a new 1H corresponding to the proton in the position 5 of the newly formed thiazole ring (**C2**–**8**) or 3H singlet corresponding to the methyl group in **C1**.

The IR spectra of the final compounds **C1**–**8** showed the presence of a sharp signal between 3111–3103 cm^−1^ characteristic of the νC5-H stretching vibration from the thiazole ring, as well as absorbance bands characteristic of aromatic rings. Specific signals for each final compound can also be identified, such as a 2920 cm^−1^ νC-H alkane stretching vibration for **C1**, two strong signals at 1519 and 1346 cm^−1^ characteristic to the asymmetric and symmetric stretching vibration due to the N-O bonds from the nitro group in **C3**, 1251 cm^−1^ and 1030 cm^−1^ bands characteristic of the asymmetric and symmetric stretching vibrations due to the C-O bonds in the ether group in **C4**, a strong absorption band at 2225 cm^−1^ due to the nitrile group in **C5**, and a high intensity absorption band at 826 cm^−1^ characteristic of the νC-Cl in **C8**. Compound **C7** showed a wide absorption band centred at 3443 cm^−1^ corresponding to the O-H stretching vibration from the phenol group, two strong absorption bands at 3326 and 3230 cm^−1^ due to the νN-H vibrations from the amide, a high intensity absorption band at 1666 cm^−1^ characteristic of the C=O bond, and a bending signal at 1619 cm^−1^ due to the N-H bond.

Mass spectra was recorded for the intermediates as well as for all final compounds and revealed the molecular ion peaks as expected from their formulas.

### 2.2. Biological Assays

The antimicrobial activity was evaluated using five Gram-positive bacterial strains and two Gram-negative bacterial strains.

#### 2.2.1. Antimicrobial Activity—Initial In Vitro Qualitative Screening Study

The results of the initial qualitative antimicrobial screening revealed that most of the tested compounds showed a good activity against most Gram-positive bacteria strains and a total lack of activity against *B. subtillis* and the Gram-negative bacteria strains, as shown in Table 1. The best antibacterial activity was against *E. faecalis* and *S. saprophyticus*, with microbial growth inhibition zone diameter values comparable with those of the standard ciprofloxacin. The solvent used, DMSO, did not produce any inhibition of bacterial growth.

#### 2.2.2. Antimicrobial Activity—In Vitro Quantitative Assay

The quantitative assay of the antimicrobial activity was performed only for the compounds that showed antimicrobial activity in the initial preliminary qualitative screening. As such, the compounds were only tested against four Gram-positive bacteria strains. The minimum inhibitory concentrations (MIC) for the compounds are shown in Table 2. They reveal modest antibacterial activity when compared with the standard ciprofloxacin. Direct antibacterial activity against *S. aureus* ATCC 6538 is not significant while against *S. aureus* BAA1026 compound **C4** has a modest activity (32 μg/mL). The strongest antibacterial activity was noticed against *E. faecalis* and *S. saprophyticus*, although it is generally modest when compared with the standard. The lowest MIC, 2 μg/mL, was determined for the chloro-derivative **C8** against *S. saprophyticus*, and it is the only antibacterial activity comparable with that of the standard.

#### 2.2.3. Anti-Biofilm Activity Assay

Biofilm formation was used as a measure of Sortase A inhibition potential, as it is generally accepted that biofilm formation is strictly dependent on the many molecules anchored by sortases at the surface of the cells. The anti-biofilm activity evaluation was performed against all bacterial species: Gram-positive and Gram-negative. Results, presented in Table 3, showed that the synthesized compounds are not active against Gram-negative bacteria biofilm formation. In the same time, when analyzing effects against Gram-positive bacteria, the effect against *E. faecalis* was prominent with MBEC values between 2–16 μg/mL. A modest activity was observed for compounds **C4**–**7** against *S. aureus* BAA1026.

### 2.3. Molecular Docking

Because of the high interest in antibacterial agents active against *Staphylococcus aureus,* a highly pathogenic, virulent and drug resistant bacteria, the catalytic process of sortases is best characterized for Sa-SrtA. Structural characterisation studies have shown that Sa-SrtA adopts an eight-stranded β-barrel structure that houses there essential active site residues: His120, Cys184 and Arg197. Transpeptidation proceeds by the binding of the LPXTG sorting signal to a large groove adjacent to the active site, through an induced-fit mechanism [6,33]. The active site thiol of Cys184 then nucleophilically attacks the carbonyl group of the substrate threonine residue forming a transient intermediate stabilized by Arg197. This intermediate rearranges into a thioacyl enzyme-substrate complex with the cleavage of the theronine-glycine peptide bond. The terminal amine group from the pentaglycine branch of lipid II then nuclepphilically attacks the carbonyl moiety of the theronine, thus completing the transfer of the initial LPXTG signal protein to the cell wall [6].

Due to the structural similarity of the active site of different sortases A, it is assumed that this catalytic process is similar for most sortases A. A phylogenetic analysis of SrtA reveals that these enzymes are closely related with various degrees of homology [17,34].

The tested compounds were docked into the catalytic site of *E. faecalis* Sortase A and *S. aureus* Sortase A enzymes. The predicted best binding affinity of conformation of each compound to the catalytic site of enzymes and the inhibition constant (*K_i_*) of the best pose are presented in Table 4. Inhibition constant (*K_i_*) was calculated based on the computed binding affinity energy (∆G) using the formula: $K_{i}=e^{\frac{∆G\times 1000}{R\times T}}$, where R represents the Regnault constant = 198,719 and T = 298.15 K.

#### 2.3.1. Sequence Alignment and Validation of the Generated Model for *E. faecalis* ATCC 29212 Sortase A

**Figure 3:**
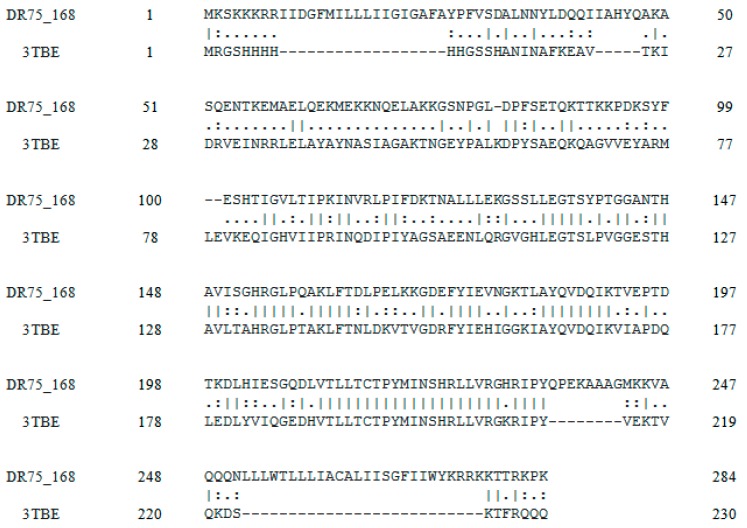
Sequence alignment between *E. faecalis* Sortase A (DR75_168) and the template structure (3TBE).

Because no three dimensional structure corresponding to Sortase A from *E. faecalis* ATCC 29212 was available in Protein Data Bank, a homology modelling operation was performed. Considering the DR75_168 sequence, that represents the primary amino acid sequence of the *E. faecalis* ATCC 29212 Sortase A target, a sequence alignment process was performed. This process provided PDB 3TBE (X-ray structure, 2.85 Ǻ resolution) as a good template for modelling, with 53.46% identity match. In addition, a supplementary alignment was performed on EMBOSS Stretcher. The comparison of template and target sequence revealed that many amino acids seem to be conserved. One to five amino acids groups randomly appear to be conserved and two large blocks with a high degree of conservation were found between 163–170, 190–201 amino acid positions, as shown in Figure 3.

**Figure 4:**
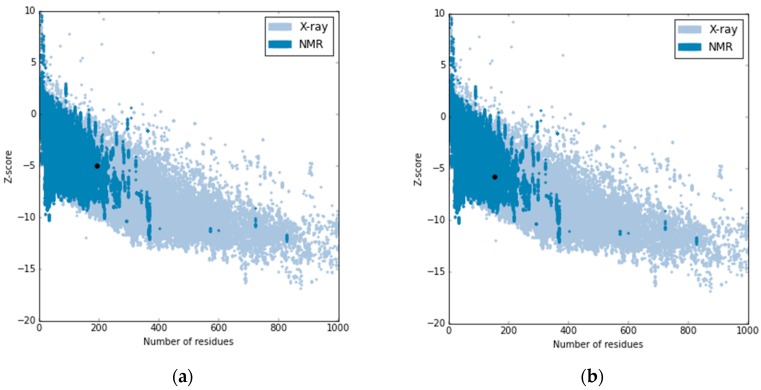
The plot representing *Z* score for the template (3TBE) (**a**) and for the new generated Sortase A for *E. faecalis* EfSRT_29212 (**b**).

Root mean square deviation (RMSD) between the template and newly generated Sortase A structure (further referred to as EfSRT_29212) structures was found to be equal to 0.129 Ǻ. *z*-Score computed for the template was found to be equal to −4.96 kcal/mol while the one for the EfSRT_29212 structure was found to be equal to −5.8 kcal/mol, as shown in Figure 4.

In the Ramachandran plot, 92.9% of the amino acid residues were found in the favoured region, 4.5% of the residues were found in the allowed region while only 2.6% of the residues were found in the disallowed region. Based on these data, the constructed structure of Sortase A of *E. faecalis* EfSRT_29212 is considered qualitative.

**Figure 5:**
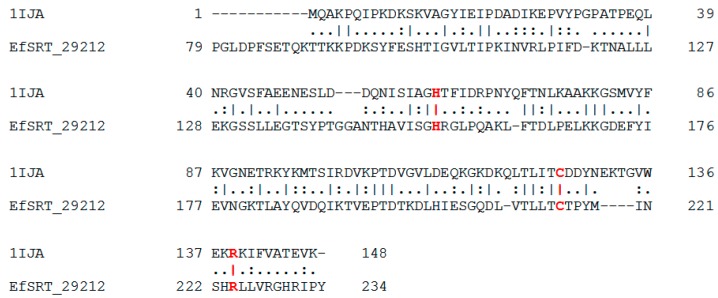
Sequence alignment of the *E. feacalis* Sortase A (EfSRT_29212) and the *S. aureus* Sortase A (PDB 1IJA). The catalytic amino acid residues are depicted in red.

*E. feacalis* Sortase A EfSRT_29212 is made of 156 amino acids while *S. aureus* Sortase A contains 148 amino acids. Sequence alignment of the two enzymes is depicted in Figure 5. Secondary structure of both enzymes of *E. faecalis* and *S. aureus* contains two β-sheets one above the other, made of four strands each, one of the strands being common.

In addition, the secondary structure of the Sortase A enzyme of *E. faecalis* has two α-helices (Thr35-Asn40 and Pro68-Ala78). Close to it, a supplementary strand (Val43-Phe45) is found, partially enlarging to the side one of the β-sheets. More than that, a small β-sheet (Tyr129-Asn130 and Val135-Glu137) is present, creating a distal region in the protein close to the catalytic site of the enzyme, which is important for binding the potential ligands.

The secondary structure of the Sortase A enzyme of *S. aureus* (PDB 1IJA) has three small α-helices and a long loop in the N-terminus part of the protein (Pro79-Gly105), part of it (Asp82-Lys89) surrounding the catalytic site of the enzyme and a smaller one (His153-Asp164). Located near the catalytic site are one of the helices (Ala124-Glu128) and two strands (Thr146-Gly152 and Asp208-Asn221). The structures alignment process found 38 amino acids identical (23.3%), 65 amino acids similar (39.9%) and 22 gaps (13.5%). This two proteins have some degree of similarity, but they have significant differences that are likely to greatly influence their properties.

#### 2.3.2. Binding Mode of the **C1**–**8** 2-Phenylthiazoles to the Catalytic Site of Sortases

The analysis of the proposed binding mode of the investigated compounds to the active sites of the two sortases revealed a totally different interaction manner. When taking into consideration the binding energy and subsequent inhibition constant *K_i_* values obtained for corresponding compounds, it became obvious that the binding to the Sortase A of *E. faecalis (*EfSRT_29212) is far superior to the binding towards the *S. aureus* Sortase A (PDB 1IJA), as shown in Table 4.

Moreover, the overall binding potential to EfSRT_29212 is greater than the overall binding potential to PDB 1IJA. This is supported by the fact that the lowest value predicted for the binding energies corresponding to the **C1**–**8**: EfSRT_29212 complexes (−9.46 kcal/mol for **C2**) is more elevated than highest value predicted for the binding energies corresponding to the **C1**–**8**: PDB 1IJA complexes (−9.00 kcal/mol for **C3**).

Considering the top binding conformations of **C1**–**8** to the active site of the Sortase A of *E. faecalis* (EfSRT_29212) we further focused on specific AA binding interactions. The main AA-inhibitor interactions and the predicted bond length values for the top binding conformation are presented in Table 5.

**Figure 6:**
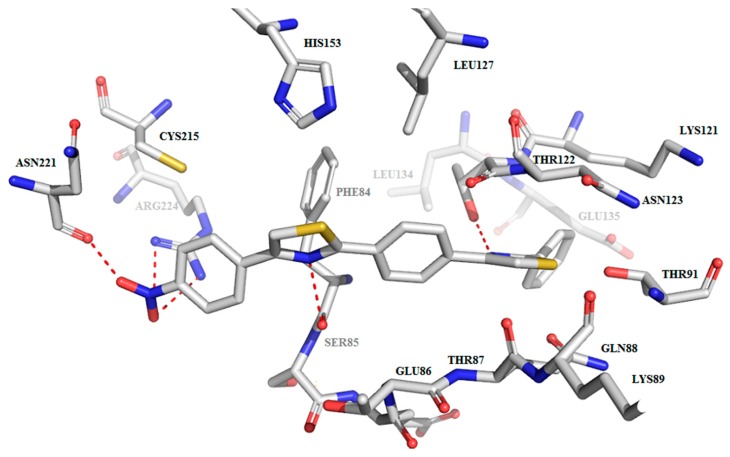
Compound **C3**’s predicted binding mode to the active site of EfSRT_29212 *E. faecalis* Sortase A. Active site and the ligands are depicted as sticks. The hydrogen bonds are depicted as dashed redlines.

The binding pattern generally involves hydrogen bonds between the thiazole nucleus nitrogen (N1 or N2) atom and the hydroxyl group from Thr122 and polar contacts between thiazole nucleus nitrogen (N1 or N2) atom and the amide carbonyl group from Thr122, as shown in Figure 6.

**Figure 7:**
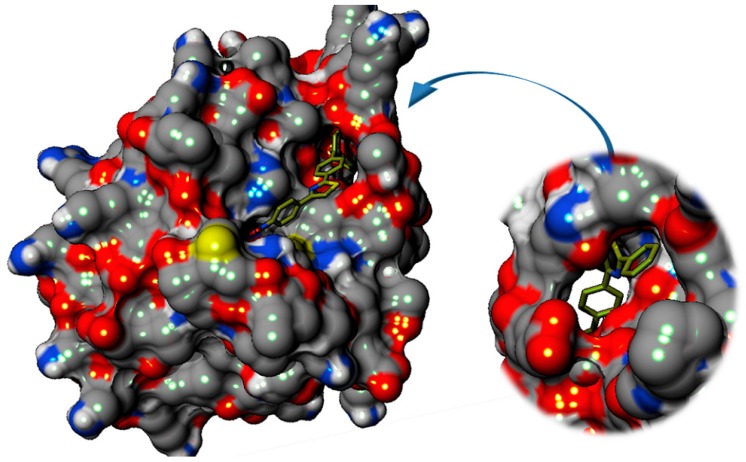
3D-surface representation of the predicted binding mode of **C1**–**8** to the active site of *E. faecalis* Sortase A (EfSRT_29212). All compounds cause a steric hindrance that prevents access to the catalytic AA triad while binding is strengthen by the two distal claws that enclose the compounds.

Furthermore, the binding affinity is augmented by a supplementary interaction that involves 2 claws that enclose the docked compounds. The claws are made up of Lys121, Thr122, Asn123 on one side and Glu86, Thr87, Gln88, Lys89, Thr90, Thr91 on the other side, as depicted in Figure 7.

Additional particular bonding is possible in the case of the compounds that have a polar substituent on the third phenyl ring. For example, due to the nitro substituent compound **C3** is able to also interact via hydrogen bonds with the guanidine moiety from Arg224 and the –NH_2_ from the amide group of Asn 221(see Figure 6). Compound **C3** seems to bind to the active site of Sortase A of *E. faecalis* (EfSRT_29212) by anchoring to the Asn221, Arg224, Ser85 and Thr 122 amino acid residues. These interactions are polar contacts that assure the binding of **C3** at the level of the catalytic site’s cavity, as depicted in Figure 6 and Figure 7. This binding causes a steric hindrance that prevents access of the substrate to the His153-Cys215-Arg224 catalytic triad.

Similarly, compound **C4** is able to form hydrogen bonds between the methoxy substituent and the guanidine moiety from Arg224. A specific interaction is possible for the salicylamide derivative **C7** which is capable of hydrogen bond interaction with the carboxyl groups of Glu135 and Asp82.

These potential interactions highlight the fact that most compounds, except **C3** and **C4**, lack direct interaction with the His153-Cys215-Arg224 catalytic triad, thus supporting the claim of a non-competitive inhibition mechanism.

The predicted binding affinities of **C1**–**8** to *S. aureus* Sortase A (PDB 1IJA) are weaker than the binding to *E. faecalis* Sortase A (EfSRT_29212). The active site of PDB 1IJA is characterised by a more lipophilic lining of the pocket caused by the dominance of lipophilic AA residues as shown in Table 6. In the same time, the site has fewer hydrogen bonds donors and fewer hydrogen bond acceptors, while the internal surface and volume of the active site pocket are smaller than those from EfSRT_29212.

**Figure 8:**
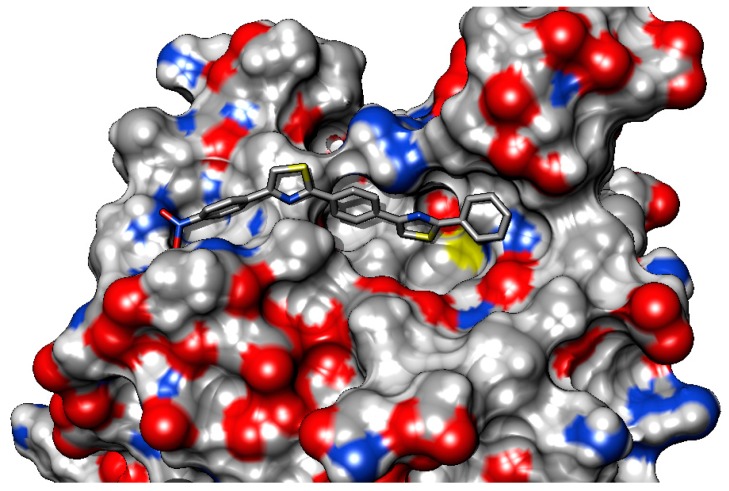
3D-surface representation of the predicted binding mode of **C1**–**8** to the active site of *S. aureus* Sortase A (PDB 1IJA). The small active site pocket does not seem able to accommodate the pentacyclic compounds.

These characteristics render the active site of *S. aureus* Sortase A less “druggable”, especially when considering the fact that the tested compounds are lengthy molecules. The molecular scaffold of our derivatives does not fit into this active site mainly because due to their length, only part of the molecule can be accommodated within the active site, as depicted in Figure 8.

Another inconvenience is represented by the presence of the Trp136 residue in the proximity of the catalytic site that hinders the access of the compounds towards the catalytic triad His62-Arg139-Cys126 [5].

**Figure 9:**
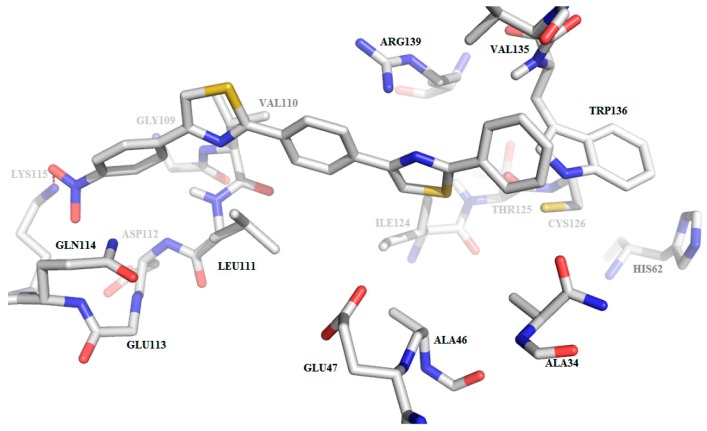
Compound **C3**’s predicted binding mode to the active site of *S. aureus* Sortase A (PDB 1IJA). Active site and the ligands are depicted as sticks. No hydrogen bonds are predicted; one polar contact (red dashes) between Lys 115 and the nitro group is anticipated.

When considering the binding profile of compound **C3**, shown in Figure 9, it is apparent that due to the active site characteristics only one polar interaction is possible (between the nitro group of C3 and the terminal amine group from Lys115). This enables a very weak anchoring process with no interactions with the AA residues of the catalytic site.

## 3. Materials and Methods

### 3.1. General Information

Chemicals used for the synthesis, isolation and purification were purchased from Merck (Darmstadt, Germany), Sigma-Aldrich (Taufkirchen, Germany) and Alfa Aesar (Karlsruhe, Germany). All chemicals were of analytical reagent grade purity.

Thin-layer chromatography was performed on silica gel sheets, with UV-light visualization. The melting points are uncorrected and were obtained by using an Electrothermal 9100 melting point apparatus (Cole-Parmer, Stone, Staffordshire, UK). IR spectra were recorded on a FT/IR 6100 spectrometer (Jasco, Cremella, Italy) after compression in anhydrous KBr pellets under vacuum. Water and carbon dioxide signals were removed from the recorded IR spectrum using the computer interface software Spectra Manager and assignment of signals was assisted by Know It All 7.8 by Bio-Rad Laboratories (Hercules, CA, USA).

MS spectra were obtained by using a Mat 111, 70 eV instrument (Varian, Bremen, Germany) by directly introduction of the solid samples. The ^1^H-NMR and ^13^C-NMR were recorded on an Avance NMR spectrometer (Bruker, Karlsruhe, Germany) operating at 500 MHz or 125 MHz, in DMSO-*d*_6_ as solvent. Chemical shift values are reported in δ units, relative to TMS as internal standard. All spectral data were in accordance with the proposed chemical structures. Elemental analysis was performed by Vario El CHNS analyzer (Hanau, Germany). The results obtained for all synthesized compounds were in agreement with the calculated values ±0.4%.

Intermediate compounds **1** and **2** as well as final compounds **C2**, **C3** and **C8** have been previously reported [35] and have been re-synthesized as a part of a series based on the generated scaffold.

### 3.2. Chemistry

Synthesis of *4-(2-phenylthiazol-4-yl)benzonitrile* (**1**). A mixture of thiobenzamide (10 mmol) and the 4-(2-bromoacetyl)benzonitrile (10 mmol) was dissolved in anhydrous acetone (50 mL) and stirred at room temperature for 24 h. The resulting solid was filtered and washed with a solution of NaHCO_3_ 5% until free of acid. The solid was then recrystallized from methanol to yield the pure compound.

*4-(2-Phenylthiazol-4-yl)benzonitrile* (**1**). White powder. m.p. 137 °C. IR (KBr) ν_max_ cm^−1^: 3103 (C-H thiazole str), 3061 (C-H str arom), 2224 (str CN), 1606, 1575, 1506, 1438 (Ar ring str), 976 (C-H bend arom), 852 (C-H def arom). ^1^H-NMR (DMSO-*d*_6_, ppm): δ 8.4 (s, 1H, H-thiazole), 8.10 (m, 2H, Ph2), 8 (m, 2H, Ph2), 7.9 (m, 2H, Ph1), 7.55 (m, 3H, Ph1). ^13^C-NMR (DMSO-*d*_6_) δ (ppm): 118.6 CN, 158.62 (thiazole C_2_), 148.76 (thiazole C_4_), 129.12 (thiazole C_5_), 128.2–131.3 (12 C, 2 phenyl rings). Anal. calcd. (%) for C_16_H_10_N_2_S (262.06): C, 73.26; H, 3.84; N, 10.68; S, 12.22. Found: C, 73.21; H, 3.89; N, 10.65; S, 12.25. MS (EI, 70 eV): *m*/*z* 262 (M^+^).

Synthesis of the *4-(2-phenylthiazol-4-yl)benzothioamide* (**2**). A solution of 4-(2-phenylthiazol-4-yl)benzonitrile (8 mmol) in ethanol (10 mL) and triethylamine (1 mL) was maintained at room temperature while hydrogen sulfide gas was bubbled into the solution for 8 h. The reaction mixture was then poured in cold water and the solid formed was filtered out, washed with water and recrystallized from ethanol.

*4-(2-Phenylthiazol-4-yl)benzothioamide* (**2**). Light-yellow powder. m.p. 179 °C. IR (KBr) ν_max_ cm^−1^: 3408, 3384 (N-H str), 3104 (C-H thiazole str), 3086 (C-H str arom), 1625 (N-H bend), 1603, 1570, 1506, 1459 (Ar ring str), 1133 (C=S str), 975 (C-H bend arom), 850 (C-H def arom). ^1^H-NMR (DMSO-*d*_6,_ ppm): δ 8.55 (s, 2H, thioamide), 8.45 (s, 1H, H-thiazole), 8 (m, 2H, Ph2), 7.9 (m, 2H, Ph1), 7.55 (m, 3H, Ph1), 7.45 (m, 2H, Ph2). ^13^C-NMR (DMSO-*d*_6_) δ (ppm): 188.2 C=S, 158.92 (thiazole C_2_), 148.96 (thiazole C_4_), 128.38 (thiazole C_5_), 128.2–143.3 (12 C, 2 phenyl rings). Anal. calcd. (%) for C_16_H_12_N_2_S_2_ (296.04): C, 64.83; H, 4.08; N, 9.45; S, 21.64. Found: C, 64.88.21; H, 4.03; N, 9.41; S, 21.69. MS (EI, 70 eV): *m*/*z* 296 (M^+^).

General Procedure for the Synthesis of the 4-Aryl-2-(4-(2-phenylthiazol-4-yl)phenyl)thiazoles (**C1**–**8**). A mixture of 4-(2-phenylthiazol-4-yl)benzothioamide (1 mmol) and the corresponding α-bromoketone (1 mmol) was dissolved in anhydrous acetone (5 mL) and stirred at room temperature for 24 h. The resulting solid was filtered and washed with a solution of NaHCO_3_ 5% until free of acid. The compounds were then recrystallized from methanol to yield the pure compounds.

*5-Methyl-4-phenyl-2-(4-(2-phenylthiazol-4-yl)phenyl)*thiazole (**C1**). Off-white powder. m.p. 235 °C. IR (KBr) ν_max_ cm^−1^: 3106 (C-H thiazole str), 3080 (C-H str arom), 2920 (C-H str CH_3_), 1602, 1572, 1503, 1439 (Ar ring str), 978 (C-H bend arom), 851 (C-H def arom). ^1^H-NMR (DMSO-*d*_6_, ppm): δ 8.33 (s, 1H, H-thiazole), 8.23 (m, 2H, Ph2), 8.18 (m, 2H, Ph2), 8.05–7.5 (m, 10H, Ph1,3), 2.75 (s, 3H, CH_3_). ^13^C-NMR (DMSO-*d*_6_) δ (ppm): 166.32 (thiazole-1 C_2_), 158.5 (thiazole-2 C_2_), 152.96 (thiazole-1 C_4_), 152.1 (thiazole-2 C_4_), 129.8 (thiazole-1 C_5_), 120.81 (thiazole-2 C_5_), 17.45 (CH_3_ thiazole), 126–130.9 (18C, 3 phenyl rings). Anal. calcd. (%) for C_25_H_18_N_2_S_2_ (410.09): C, 73.14; H, 4.42; N, 6.82; S, 15.62. Found: C, 73.01; H, 4.45; N, 6.89; S, 15.65. MS (EI, 70 eV): *m*/*z* 410 (M^+^).

*2-Phenyl-4-(4-(4-phenylthiazol-2-yl)phenyl)thiazole* (**C2**). Off-white powder. m.p. 205 °C. IR (KBr) ν_max_ cm^−1^: 3108 (C-H thiazole str), 3069 (C-H str arom), 1599, 1576, 1502, 1458 (Ar ring str), 980 (C-H bend arom), 851 (C-H def arom). ^1^H-NMR (DMSO-*d*_6_, ppm): δ 8.53 (s, 1H, H-thiazole), 8.35 (s, 1H, H-thiazole), 8.25 (m, 2H, Ph2), 8.15 (m, 2H, Ph2), 8.02–7.4 (m, 10H, Ph1,3). ^13^C-NMR (DMSO-*d*_6_) δ (ppm): 166.1 (thiazole-1 C_2_), 159 (thiazole-2 C_2_), 152.66 (thiazole-1 C_4_), 152.3 (thiazole-2 C_4_), 129.2 (thiazole-1 C_5_), 121.11 (thiazole-2 C_5_), 126.4–130 (18C, 3 phenyl rings). Anal. calcd. (%) for C_24_H_16_N_2_S_2_ (396.53): C, 72.70; H, 4.07; N, 7.06; S, 16.17. Found: C, 72.55; H, 4.12; N, 7.13; S, 16.2. MS (EI, 70 eV): *m*/*z* 396 (M^+^).

*4-(4-Nitrophenyl)-2-(4-(2-phenylthiazol-4-yl)phenyl)*thiazole (**C3**). Yellow powder. m.p. 238 °C. IR (KBr) ν_max_ cm^−1^: 3110 (C-H thiazole str), 3081 (C-H str arom), 1597, 1577 (Ar ring str), 1519 (N-O NO_2_ str), 1448 (Ar ring str), 1346 (N-O NO_2_ str), 980 (C-H bend arom), 842 (C-H def arom). ^1^H-NMR (DMSO-*d*_6_, ppm): δ 8.6 (s, 1H, H-thiazole), 8.4 (s, 1H, H-thiazole), 8.33 (dd, 2H, Ph-NO_2_), 8.25 (dd, 2H, Ph-NO_2_), 8.2 (m, 2H, Ph2), 8.10 (m, 2H, Ph2), 8 (m, 2H, Ph1), 7.55 (m, 3H, Ph1). ^13^C-NMR (DMSO-*d*_6_) δ (ppm): 165.82 (thiazole-1 C_2_), 158.7 (thiazole-2 C_2_), 153.26 (thiazole-1 C_4_), 151.8 (thiazole-2 C_4_), 127.8 (thiazole-1 C_5_), 119.81 (thiazole-2 C_5_), 126–147.2 (18C, 3 phenyl rings). Anal. calcd. (%) for C_24_H_15_N_3_O_2_S_2_ (441.06): C, 65.29; H, 3.42; N, 9.52; S, 14.52. Found: C, 65.33; H, 3.40; N, 9.54; S, 14.49. MS (EI, 70 eV): *m*/*z* 441(M^+^).

*4-(4-Methoxyphenyl)-2-(4-(2-phenylthiazol-4-yl)phenyl)thiazole* (**C4**). Yellowish—brown powder. m.p. 223 °C. IR (KBr) ν_max_ cm^−1^: 3109 (C-H thiazole str), 3057 (C-H str arom), 1608, 1578, 1503, 1460 (Ar ring str), 1251 (C-O-C assym str), 1030 (C-O-C sym str), 981 (C-H bend arom), 838 (C-H def arom). ^1^H-NMR (DMSO-*d*_6_, ppm): δ 8.65 (s, 1H, H-thiazole), 8.38 (s, 1H, H-thiazole), 8.3 (m, 2H, Ph2), 8.20 (m, 2H, Ph2), 8 (m, 2H, Ph-OCH_3_) 8.06 (m, 2H, Ph1), 7.60 (m, 3H, Ph1), 7.05 (dd, 2H, Ph-OCH_3_), 3.85 (s, 3H, OCH_3_). ^13^C-NMR (DMSO-*d*_6_) δ (ppm): 166.92 (thiazole-1 C_2_), 157.9 (thiazole-2 C_2_), 152.66 (thiazole-1 C_4_), 152.18 (thiazole-2 C_4_), 128.2 (thiazole-1 C_5_), 120.21 (thiazole-2 C_5_), 56.92 (OCH_3_), 126–159.04 (18C, 3 phenyl rings). Anal. calcd. (%) for C_25_H_18_N_2_OS_2_ (426.09): C, 70.39; H, 4.25; N, 6.57; S, 15.03. Found: C, 70.44; H, 4.21; N, 6.59; S, 15.13. MS (EI, 70 eV): *m*/*z* 426 (M^+^).

*4-(2-(4-(2-Phenylthiazol-4-yl)phenyl)thiazol-4-yl)benzonitrile* (**C5**). Yellow powder. m.p. 242 °C. IR (KBr) ν_max_ cm^−1^: 3110 (C-H thiazole str), 3061 (C-H str arom), 2225 (str CN), 1605, 1575, 1503, 1448 (Ar ring str), 980 (C-H bend arom), 844 (C-H def arom). ^1^H-NMR (DMSO-*d*_6_, ppm): δ 8.55 (s, 1H, H-thiazole), 8.45 (dd, 2H, Ph-CN), 8.30 (s, 1H, H-thiazole), 8.25 (m, 2H, Ph2), 8.15 (m, 2H, Ph2), 7.9 (m, 2H, Ph1), 7.77 (dd, 2H, Ph-CN), 7.50 (m, 3H, Ph1). ^13^C-NMR (DMSO-*d*_6_) δ (ppm): 165.62 (thiazole-1 C_2_), 157.85 (thiazole-2 C_2_), 152.91 (thiazole-1 C_4_), 151.81 (thiazole-2 C_4_), 128.8 (thiazole-1 C_5_), 118.6 CN, 119.91 (thiazole-2 C_5_), 126–130.8 (18C, 3 phenyl rings). Anal. calcd. (%) for C_25_H_15_N_3_S_2_ (421.07): C, 71.23; H, 3.59; N, 9.97; S, 15.21. Found: C, 71.4; H, 3.64; N, 9.70; S, 15.26. MS (EI, 70 eV): *m*/*z* 421 (M^+^).

*4-(Naphthalen-1-yl)-2-(4-(2-phenylthiazol-4-yl)phenyl)thiazole* (**C6**). Off-white powder. m.p. 237 °C. IR (KBr) ν_max_ cm^−1^: 3109 (C-H thiazole str), 3061 (C-H str arom), 1603, 1571, 1504, 1455 (Ar ring str), 979 (C-H bend arom), 839 (C-H def arom). ^1^H-NMR (DMSO-*d*_6_, ppm): δ 8.6 (s, 1H, H-thiazole), 8.42 (s, 1H, H-thiazole), 8.25 (m, 2H, Ar2), 8.15 (m, 2H, Ph2), 8.06–8.03 (m, 2H, naph), 7.9 (m, 2H, Ph1), 7.56–7.53 (m, 4H, naph), 7.45 (m, 3H, Ph1). ^13^C-NMR (DMSO-*d*_6_) δ (ppm): 166.32 (thiazole-1 C_2_), 158.5 (thiazole-2 C_2_), 152.96 (thiazole-1 C_4_), 152.1 (thiazole-2 C_4_), 128.8 (thiazole-1 C_5_), 120.61 (thiazole-2 C_5_), 126–140.6 (22 C, 2 phenyl and 1 naphtyl rings). Anal. calcd. (%) for C_28_H_18_N_2_S_2_ (446.09): C, 75.30; H, 4.06; N, 6.27; S, 14.36. Found: C, 75.26; H, 4.18; N, 6.31; S, 14.25. MS (EI, 70 eV): *m*/*z* 446 (M^+^).

*2-Hydroxy-5-{2-[4-(2-phenyl-1,3-thiazol-4-yl)phenyl]-1,3-thiazol-4-yl}benzamide* (**C7**) Light-grey. m.p. 225 °C. IR (KBr) ν_max_ cm^−1^: 3443 (O-H str phenol), 3326, 3230 (N-H str amide), 3108 (C-H thiazole str), 3059 (C-H str arom), 1666 (C=O str amide), 1619 (N-H bend amide), 1601, 1505, 1447 (Ar ring str), 983 (C-H bend arom), 840 (C-H def arom). ^1^H-NMR (DMSO-*d*_6_, ppm): δ 13.2 (s, 1H, OH), 8.63 (s, 1H, H-thiazole), 8.56 (d, 1H, Ph3), 8.35 (s, 1H, H-thiazole), 8.23 (m, 2H, Ph2), 8.18 (m, 2H, Ph2), 8.13 (m, 1H, Ph3), 8.06 (m, 2H, Ph1), 8.02 (s, 2H, CONH_2_), 7.55 (m, 3H, Ph1), 7.02 (d, 1H, Ph3). ^13^C-NMR (DMSO-*d*_6_) δ (ppm): 167.66 (amide C=O), 166.32 (thiazole-1 C_2_), 158.5 (thiazole-2 C_2_), 152.96 (thiazole-1 C_4_), 152.1 (thiazole-2 C_4_), 129.8 (thiazole-1 C_5_), 120.41 (thiazole-2 C_5_), 126-159.6 (18 C, 3 phenyl rings). Anal. calcd. (%) for C_25_H_17_N_3_O_2_S_2_ (455.08): C, 65.91; H, 3.76; N, 9.22; S, 14.08. Found: C, 65.8; H, 3.6; N, 9.3; S, 14.4. MS (EI, 70 eV): *m*/*z* 455 (M^+^).

*4-(4-Chlorophenyl)-2-(4-(2-phenylthiazol-4-yl)phenyl)thiazole* (**C8**) Off-white powder. m.p. 232 °C. IR (KBr) ν_max_ cm^−1^: 3111 (C-H thiazole str), 3059 (C-H str arom), 1597, 1572, 1503, 1462 (Ar ring str), 981 (C-H bend arom), 843 (C-H def arom), 826 (C-Cl str). ^1^H-NMR (DMSO-*d*_6_, ppm): δ 8.56 (s, 1H, H-thiazole), 8.35 (s, 1H, H-thiazole), 8.30 (m, 2H, Ph2), 8.20 (m, 2H, Ph2), 7.9 (m, 2H, Ph1), 7.60 (dd, 2H, Ph-Cl), 7.55 (dd, 2H, Ph-Cl), 7.45 (m, 3H, Ph1). ^13^C-NMR (DMSO-*d*_6_) δ (ppm): 165.32 (thiazole-1 C_2_), 159.5 (thiazole-2 C_2_), 151.96 (thiazole-1 C_4_), 151.1 (thiazole-2 C_4_), 128.38 (thiazole-1 C_5_), 120.81 (thiazole-2 C_5_), 126–132.89 (18 C, 3 phenyl rings). Anal. calcd. (%) for C_24_H_15_ ClN_2_S_2_ (430.04): C, 66.89; H, 3.51; N, 6.50; S, 14.88. Found: C, 66.80; H, 3.55; N, 6.52; S, 14.91. MS (EI, 70 eV): *m*/*z* 430 (M^+^).

### 3.3. Biological Assays

The antimicrobial activity assessment was performed using 3 different methods: an initial qualitative screening, a quantitative assay and also an assessment of the antibiofilm activity that aims at determining the antipathogenic potential. The quantitative assays were performed only for the compounds that showed a promising activity in the initial qualitative screening.

The antibiofilm activity was assessed for all compounds due to the fact that the mechanisms for antibiofilm activity can be different and independent from those involved in bactericidal activity.

#### 3.3.1. Antimicrobial Activity—Initial In Vitro Qualitative Screening Study

The antimicrobial activity of the synthesized compounds was assayed on Gram-negative bacteria (*Escherichia coli* ATCC 8739, *Pseudomonas aeruginosa* ATCC 27853) and Gram-positive bacteria (*Enterococcus faecalis* ATCC 29212, *Staphylococcus aureus* ATCC 6538, *Staphylococcus aureus* BAA 1026, *Staphylococcus saprophyticus* ATCC 15305, *Bacillus subtilis* ATCC 6633). Microbial suspensions of 1.5 × 10^8^ CFU mL^−1^ (0.5 McFarland density) obtained from 15 to 18 h bacterial cultures developed on solid media were used. The antimicrobial activity was tested on Mueller–Hinton Agar (MHA) medium. The analyzed compounds and the reference substance, ciprofloxacin, were solubilized in DMSO. The starting stock solution was of 10 mg/mL concentration. The qualitative screening was performed by an adapted disc diffusion method as previously reported [36]. The inoculated plates were incubated for 24 h at 37 °C for bacterial strains. Growth inhibition zones diameters were measured (mm) as an assessment of antimicrobial activity.

#### 3.3.2. Antimicrobial Activity—In Vitro Quantitative Assay

The quantitative assay of the antimicrobial activity was performed by liquid medium microdilution method in 96 multi-well plates. Two-fold serial dilutions of the compounds solutions (ranging between 1000 μg and 2 μg mL^−1^) were performed in a 200 μL volume of broth, and each was well seeded with 50 μL microbial inoculum. Reference antimicrobial agents were used as standard (ciprofloxacin). Culture positive controls (wells containing culture medium seeded with the microbial inoculum) were used. The influence of the DMSO solvent was also quantified in a series of wells containing DMSO, diluted accordingly with the dilution scheme. The plates were incubated for 24 h at 37 °C for bacterial strains. The minimal inhibitory concentration (MIC) values were considered as the lowest concentration of the tested compound that inhibited the growth of the microbial cultures, as compared to the positive control, revealed by a decreased value of absorbance at 600 nm (Apollo LB 911 ELISA Absorbance Reader, Berthold Technologies, Bad Wildbad, Germany) [28,29,37].

#### 3.3.3. Anti-Biofilm Activity Assay

For the evaluation of the influence of the tested compounds on the ability of the tested bacterial strains to colonize the inert substratum, a microtiter plate method was used. The microplates used for the MIC assay were emptied and washed three times by phosphate buffered saline. The biofilm formed on the plastic wells wall was fixed for 5 min with cold methanol, coloured for 15 min by violet crystal solution and resuspended with a 33% acetic acid solution. Cell density was measured by reading the optical density of the coloured solution at 490 nm. The minimal biofilm eradication concentration (MBEC) values were considered as the lowest concentration of the tested compound that inhibited the development of biofilm on the plate wells [37].

### 3.4. Molecular Docking Study

In order to understand the mechanism of action of the synthesized 2-phenylthiazole compounds a molecular docking study was performed using AutoDock 4.2 [38]. Our compounds were tested against Sortase A from *E. faecalis* and Sortase A from *S. aureus*, to evaluate the binding affinity and possible interactions with the two proteins. Dataset of the 3D compounds was designed using HyperChem 6, with geometry optimization and minimized energy conformation. Further, all ligand input files preparation was performed using AutoDock Tools 1.5.6 [38]. Ligand preparation included addition of Gasteiger charges and merging non-polar hydrogen atoms. For each ligand AutoDock searched for 50 conformers. 9 × 10^4^ was set as the maximum number of evaluation, 0.02 rate of mutation, 0.8 rate of crossover and 2 Ǻ step size for translations.

The three dimensional structure of Sortase A from *E. faecalis* ATCC 29212 was built by homology modeling using Swiss-Model service, based on its 224 amino acids sequence (DR75_168) taken from KEGG (Kyoto Encyclopedia of Genes and Genomes) and PDB 3TBE as template. The obtained three dimensional structure was checked with ProSA [39,40], in order to search potential errors in prediction of the structure of the modelled protein. Numbering of amino acids in the resulted structure was left as given, first amino acid in structure EfSRT_29212 being Pro79 and the last one Tyr234. The three dimensional structure of *S. aureus* Sortase A used was PDB 1IJA.

Using AutoDock Tools, proteins were prepared for docking: addition of polar hydrogen atoms, deprotonation of carboxylic moieties and assignation of Gasteiger partial charges. Grid boxes size was defined as x = y = z = 74 Ǻ in dimension for both enzymes. Center coordinates of the cubic search space in Sortase A docking was centered to x = 21.385, y = 1.318, z = −3.296 in *E. faecalis* and x = −1.244, y = 3.910, z = 4.892 in *S. aureus*. Cartesian parameters for search space were chosen in order to include the amino acids from the catalytic site of the enzymes inside. Catalytic triad is composed by His153, Cys215 and Arg224 in *E. faecalis* Sortase A, respectively His62, Cys126 and Arg139 for *S. aureus* Sortase A. Catalytic sites were identified using BLASTP 2.6.1 [41]. A sequence comparison and alignment was performed, in order to evaluate supplementary the two studied enzymes: PDB 1IJA and EfSRT_29212. Sequence alignment of the proteins was performed using EMBOSS Stretcher 6.0, based on EBLOSUM62, with pairing output format. Analyses of binding sites of sortases A were performed using DoGSiteScorer [42]. Ramachandran plot was generated by RAMPAGE [43]. Visualization and analysis of the docking results were performed using PyMol and UCSF Chimera [44].

## 4. Conclusions

Starting from the general scaffold of sortase inhibitors obtained from the literature review [25] we synthesized and characterized eight compounds containing a phenylthiazole within a pentacyclic system. All physicochemical parameters and analysis confirmed the identity of the structures.

The antimicrobial activity assays performed showed that the compounds have a weak antimicrobial effect and as such do not markedly impact bacterial cell viability. This mild antimicrobial effect is however focused against Gram-Positive bacteria, mainly *E. faecalis* and *S. aureus*. These results are in accordance with the initial goal of obtaining Sortase inhibitors that decrease bacterial virulence but do not cause selective pressure by bacteriostatic or bactericidal effects.

When considering the anti-biofilm formation activity result showed an excellent activity against *E. faecalis* biofilm and a moderate activity against *S. aureus*. In order to understand this phenomenon molecular docking studies were performed against Sortase A from the 2 bacterial species. Results from the in silico binding prediction correlate with the in vitro anti-biofilm assays. As such, we obtained a series of new compounds capable of disturbing biofilm formation especially in the case of *E. faecalis*. This selectivity can be explained by the differences between active site conformations of the sortases from the 2 bacterial strains. Most of the compounds act as a non-competitive inhibitor of the *E. faecalis* sortase by binding to the active site and creating a steric hindrance, with little or no interaction with the catalytic residues. This binding is not favoured in the case of *S. aureus* Sortase A, which has a narrower active site that cannot accommodate our inhibitors. The importance of inhibiting Sortase A in order to inhibit biofilm formation in *E. faecalis* has been previously reported [10,11] and is confirmed by our findings.

Obtaining compounds active against *E. faecalis* is of major interest as most sortase inhibitors obtained so far focused on *S. aureus*. *E. faecalis* is a microorganism associated with endodontic infections [45], chronic periradicular lesions, endocarditis [13], nosocomial infections and potentially even colorectal neoplasm [12]. Multidrug-Resistant *E. faecalis* infections are very common [2,14] and are enabled by the biofilm formation properties of these bacteria [10,15]. In these circumstances the need for novel therapies is stringent and the use of anti-biofilm agents to decrease virulence, in a combination with known antibacterial agents, could become a feasible strategy.

## Figures and Tables

**Table 1:** The antimicrobial activity of the tested compounds expressed as microbial growth inhibition diameters (mm).

Compound	*Enterococcus faecalis* ATCC 29212	*Staphylococcus aureus* ATCC 6538	*Staphylococcus aureus* BAA 1026	*Staphylococcus saprophyticus* ATCC 15305	*Bacillus subtilis* ATCC 6633	*Escherichia coli* ATCC 8739	*Pseudomonas aeruginosa* ATCC 27853
**C1**	14	9	10	10	0	0	0
**C2**	13	8	8	11	0	0	0
**C3**	14	8	7	13	0	0	0
**C4**	12	8	8	10	0	0	0
**C5**	13	9	8	12	0	0	0
**C6**	14	10	0	0	0	0	0
**C7**	10	0	0	0	0	0	0
**C8**	12	9	10	10	0	0	0
Ciprofloxacin	15	14	15	16	14	14	16
DMSO	0	0	0	0	0	0	0

**Table 2:** The minimum inhibitory concentrations MIC (mg mL^−1^) values of the tested compounds against the tested microbial strains.

Compound	*Enterococcus faecalis* ATCC 29212	*Staphylococcus aureus* ATCC 6538	*Staphylococcus aureus* BAA 1026	*Staphylococcus saprophyticus* ATCC 15305
**C1**	0.25	>1	>1	>1
**C2**	0.125	>1	0.125	0.032
**C3**	0.062	>1	>1	0.032
**C4**	0.062	>1	0.032	0.125
**C5**	0.062	>1	>1	0.016
**C6**	0.016	>1	-	-
**C7**	0.25	-	-	-
**C8**	0.032	>1	>1	0.002
Ciprofloxacin	0.002	0.0005	0.001	0.0005

**Table 3:** The minimal biofilm eradication concentration MBEC (mg mL^−1^) values of the tested compounds against the tested microbial strains.

Compound	*Enterococcus faecalis* ATCC 29212	*Staphylococcus aureus* ATCC 6538	*Staphylococcus aureus* BAA 1026	*Staphylococcus saprophyticus* ATCC 15305	*Bacillus subtilis* ATCC 6633	*Escherichia coli* ATCC 8739	*Pseudomonas aeruginosa* ATCC 27853
**C1**	0.004	1	1	>1	1	1	1
**C2**	0.002	1	1	>1	1	1	1
**C3**	0.002	>1	>1	>1	1	>1	1
**C4**	0.004	>1	0.062	>1	1	>1	1
**C5**	0.002	1	0.25	>1	1	1	>1
**C6**	0.004	1	0.25	>1	>1	1	>1
**C7**	0.008	1	0.25	>1	>1	1	1
**C8**	0.016	>1	>1	>1	>1	1	1

**Table 4:** Binding energies and Inhibition constants for the **C1**–**8**—Sortase A complexes.

Compound	*E. faecalis* Sortase A		*S. aureus* Sortase A	
	Binding Energy (kcal/mol)	Inhibition Constant (nM)	Binding Energy (kcal/mol)	Inhibition Constant (nM)
**C1**	−9.54	101.64	−8.16	1043.84
**C2**	−9.46	116.34	−7.25	4849.25
**C3**	−10.54	18.80	−9.00	252.88
**C4**	−9.94	51.75	−7.28	4609.83
**C5**	−10.64	15.88	−8.50	588.05
**C6**	−10.56	18.17	−7.90	1618.89
**C7**	−9.67	81.62	−8.37	732.32
**C8**	−10.01	45.98	−8.61	488.41

**Table 5:**
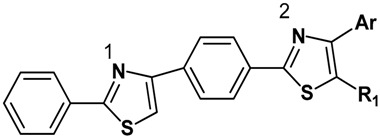
Predicted polar contacts between **C1**–**8** and Sortase A from *E. faecalis* (EfSRT_29212).

Compound	Ligand Atom ID	Interacting AA Residue	Bond Length (Å)
**C1**	N1	Thr122-OH	2.9
	N1	Thr122-C=O	3.1
**C2**	N1	Thr122-OH	3.1
**C3**	N1	Thr122-OH	2.8
	N2	Ser85-C=O	3.0
	*O*-nitro	Arg224-C=NH	2.8
	*O*-nitro	Arg224-C-NH_2_	3.5
	*O*-nitro	Asn221-C-NH_2_	2.5
**C4**	N1	Thr122-OH	2.9
	N2	Ser85-C=O	2.9
	*O*-methoxy	Arg224-C=NH	3.0
	*O*-methoxy	Arg224-C-NH_2_	3.3
**C5**	N1	Thr122-OH	2.8
	*N*-cyano	Asn221-C=O	3.5
**C6**	N1	Thr122-OH	2.9
	N1	Thr122-C=O	3.1
**C7**	N2	Thr122-OH	2.9
	N2	Thr122-C=O	3.1
	OH	Glu135-COOH	2.8
	NH2	Asp82-COOH	3.0
**C8**	N1	Thr122-OH	2.9
	N1	Thr122-OH	3.0

**Table 6:** Comparative description of the characteristic parameters of the 2 sortases A active sites.

Parameter	*E. faecalis*	*S. aureus*
Volume	580.03 Ǻ^3^	387.65 Ǻ^3^
Internal surface	1022.95 Ǻ^2^	654.68 Ǻ^2^
H bond donor AA	23	9
H bond acceptor AA	56	24
Non polar AA	29%	52%
Polar non-ionic AA	34%	14%
Cationic AA	21%	24%
Anionic AA	16%	10%
